# Urinary L-FABP Assay in the Detection of Acute Kidney Injury following Haematopoietic Stem Cell Transplantation

**DOI:** 10.3390/jpm14101046

**Published:** 2024-10-09

**Authors:** Roshni Mitra, Eleni Tholouli, Azita Rajai, Ananya Saha, Sandip Mitra, Nicos Mitsides

**Affiliations:** 1 Department of Haemato-Oncology, St Bartholomew’s Hospital, Barts Health NHS Trust, London EC1A 7BE, UK; 2 Department of Haematology, Manchester University Hospitals, Oxford Road, Manchester M13 9WL, UK; eleni.tholouli@mft.nhs.uk; 3 Research and Innovation, Manchester University Hospitals, Oxford Road, Manchester M13 9WL, UK; 4 Manchester Academy of Health Sciences, Manchester University Hospitals, University of Manchester, Manchester M13 9WL, UKsandip.mitra@mft.nhs.uk (S.M.); 5 Medical School, University of Cyprus, Nicosia 2029, Cyprus; 6 Nephrology Department, Nicosia General Hospital, Nicosia 2029, Cyprus

**Keywords:** biomarker, creatinine, urinary L-FABP, allogenic transplantation, autologous transplantation

## Abstract

**Background**: Acute Kidney Injury (AKI) is a condition that affects a significant proportion of acutely unwell patients and is associated with a high mortality rate. Patients undergoing haemopoietic stem cell transplantation (HSCT) are in an extremely high group for AKI. Identifying a biomarker or panel of markers that can reliably identify at-risk individuals undergoing HSCT can potentially impact management and outcomes. Early identification of AKI can reduce its severity and improve prognosis. We evaluated the urinary Liver type fatty acid binding protein (L-FABP), a tubular stress and injury biomarker both as an ELISA and a point of care (POC) assay for AKI detection in HSCT. **Methods**: 85 patients that had undergone autologous and allogenic HSCT (35 and 50, respectively) had urinary L-FABP (uL-FABP) measured by means of a quantitative ELISA and a semi-quantitative POC at baseline, day 0 and 7 post-transplantation. Serum creatinine (SCr) was also measured at the same time. Patients were followed up for 30 days for the occurrence of AKI and up to 18 months for mortality. The sensitivity and specificity of uL-FABP as an AKI biomarker were evaluated and compared to the performance of sCr using ROC curve analysis and logistic regression. **Results**: 39% of participants developed AKI within 1 month of their transplantation. The incidence of AKI was higher in the allogenic group than in the autologous HTSC group (57% vs. 26%, *p* = 0.008) with the median time to AKI being 25 [range 9-30] days. This group was younger (median age 59 vs. 63, *p* < 0.001) with a lower percentage of multiple myeloma as the primary diagnosis (6% vs. 88%, *p* < 0.001). The median time to AKI diagnosis was 25 [range 9–30] days. uL-FABP (mcg/gCr) at baseline, day of transplant and on the 7th day post-transplant were 1.61, 5.39 and 10.27, respectively, for the allogenic group and 0.58, 4.36 and 5.14 for the autologous group. Both SCr and uL-FABP levels rose from baseline to day 7 post-transplantation, while the AUC for predicting AKI for baseline, day 0 and day 7 post-transplant was 0.54, 0.59 and 0.62 for SCr and for 0.49, 0.43 and 0.49 uL-FABP, respectively. Univariate logistic regression showed the risk of AKI to be increased in patients with allogenic HSCT (*p* = 0.004, 95%CI [0.1; 0.65]) and in those with impaired renal function at baseline (*p* = 0.01, 95%CI [0.02, 0.54]). The risk of AKI was also significantly associated with SCr levels on day 7 post-transplant (*p* = 0.03, 95%CI [1; 1.03]). Multivariate logistic regression showed the type of HSCT to be the strongest predictor of AKI at all time points, while SCr levels at days 0 and 7 also correlated with increased risk in the model that included uL-FABP levels at the corresponding time points. The POC device for uL-FABP measurement correlated with ELISA (*p* < 0.001, Spearman ‘correlation’ = 0.54) **Conclusions**: The urinary biomarker uL-FABP did not demonstrate an independent predictive value in the detection of AKI at all stages. The most powerful risk predictor of AKI in this setting appears to be allograft recipients and baseline renal impairment, highlighting the importance of clinical risk stratification. Urinary L-FAPB as a POC biomarker was comparable to ELISA, which provides an opportunity for simple and rapid testing. However, the utility of LFABP in AKI is unclear and needs further exploration. Whether screening through rapid testing of uL-FABP can prevent or reduce AKI severity is unknown and merits further studies.

## 1. Introduction

Approximately 30,000–40,000 Haematopoietic Stem Cell Transplantations (HSCTs) are performed annually worldwide, offering a cure to many malignant and non-malignant haematological diseases [1,2]. Despite the incremental advances in outcomes following HSCT, acute renal insufficiency remains a common complication post-transplant, often negatively influencing the outcome [2,3]. Currently, the incidence of Acute Kidney Injury (AKI) varies widely between 20% and 73%. The reported incidence, timing and severity of post-transplant AKI vary according to the conditioning regime (myeloablative vs. non-myeloablative) and/or the type of HSCT (autologous or allogenic) used [1,2,3,4,5,6,7,8,9]. The frequency of AKI also increases significantly from autologous HSCT (21%) to non-myeloablative allogenic (40%) to myeloablative allogenic (69%) HSCT [10].

The current practice for diagnosing and risk stratifying AKI is based on clinical and biochemical criteria initially set by the Risk, Injury Failure Loss End-stage renal disease (RIFLE) criteria and by the Acute Kidney Injury Network (AKIN) [11] and Kidney Disease Improving Global Outcomes (KDIGOs) [12]. These are based on an assessment of the serum creatinine deviation from baseline and the measurement of urine output over time [13]. Serum creatinine is a by-product of skeletal muscle metabolism and the non-enzymatic conversion of creatine and phosphocreatine to creatinine. In health, the serum creatinine concentration is maintained in a steady state by the balance between its generation in skeletal muscle tissues and its excretion by the kidneys under a physiological glomerular filtration rate (GFR) [14]. However, creatinine is a valid measure of GFR change only in the presence of a steady state of the other variables determining its generation rate. In the presence of chronic illness or hospitalized acute illnesses with high catabolic states, loss of muscle mass, cachexia and fluid overload or interventions with powerful therapeutic agents, as in cancer therapies, creatinine values might vary significantly [15] and are not affected by kidney function alone. Crucially, the relationship between serum creatinine and GFR is not only reciprocal but also exponential, thus substantial changes in the GFR may occur despite minor changes in serum creatinine during the initial phases of kidney injury [16]. Hence, trivial increases in serum creatinine (10%) have been associated with prolonged intensive care unit (ICU) stay and increased mortality in critically ill patients with malignant disease [17]. Creatinine as such is an imperfect marker of early declines in renal function and AKI. The impact of this is more significant and apparent in at-risk populations such as the critically ill and those receiving potentially nephrotoxic therapeutic agents. Cancer patients are such a vulnerable high-risk group and, within this, recipients of HSCT form an important subgroup. Improving the risk assessment and stratification pathways for the early identification of the risk of developing AKI in these groups of patients could have a significant impact on the adverse outcomes and mortality associated with it.

Liver-type fatty acid binding protein (L-FABP) is one of the most promising biomarkers for AKI and it has been shown to be of value in the monitoring and prediction of kidney diseases [18,19,20,21,22]. Urinary L-FABP (uL-LABP) has been reported to be useful for the diagnosis and prediction of AKI in patients who have undergone cardiac surgery and have contrast-induced nephropathy [23,24]. Free fatty acids (FFAs) bound to albumin are filtered through glomeruli and reabsorbed into the proximal tubule along with albumin [25]. L-FABP transports fatty acids to mitochondria where they are metabolized by β-oxidation [26,27]. Renal proximal tubular cells under ischaemic or oxidative stress tend to downregulate this pathway, resulting in an increased fatty acid concentration [28]. Peroxidised fatty acids are cytotoxic and can lead to tubulo-interstitial damage and the deterioration of renal function [29,30]. Consequently, and through upregulation of the genetic expression of L-FABP, increased excretion of urinary L-FABP occurs. uL-FABP has been investigated both in isolation or used in conjunction with other biomarkers [31,32] in critical care [33,34], cardiac surgery [31,35,36,37], trauma [38], intravenous imaging contrast exposure [35,39,40,41], abdominal aortic aneurism [42] repair surgery, other major surgery [32,43], chemotherapy-induced kidney injury from cisplatin [44] and haemopoietic stem cell transplantation [45].

We aimed to assess the potential utility of urinary L-FABP as a biomarker for the detection of AKI in patients receiving haemopoietic stem cell transplantation utilising a semi-quantitate Point-of-Care (POC) device that could enable prompt screening of at-risk patients at the bedside.

## 2. Materials and Methods

### 2.1. Study Design and Population

The study was a prospective performance evaluation study of uL-FABP as a potential biomarker of AKI in HSCT recipients. Adult patients with the capacity to consent to receive either allogenic or autologous transplant HSCTs at one of the largest UK centres were eligible to participate in the study. They were approached during their attendance at the clinic. Potential subjects were provided with the study information at least 24 h prior to admission to hospital for treatment. Participation in the study was voluntary. Informed written consent was obtained before enrolment at the stage of initiation of the conditioning regimen for HSCT. The study received approval from the Yorkshire and Humber-Leeds West NHS Research Ethics Committee (15/YH/0516), was adopted based on the NIHR clinical research network portfolio (CRN ID: 20484) and was performed in accordance with the Declaration of Helsinki. The recruitment phase lasted 12 months.

### 2.2. Haematological Conditions and Conditioning Regimens

Patients with multiple myeloma were conditioned with Melphalan 200 mg/m^2^ intravenously (IV) in 250 mL of normal saline (dose reduction to 140 mg/m^2^ if SCr > 200 μmol/L) before HSCT.

#### 2.2.1. Patients with Lymphoma Were Conditioned with Either

(a) Etoposide 200 mg/m^2^ in 1000 mL of normal saline over 2 h and Cytosine Arabinoside 200 mg/m^2^ 12 hourly over 30 min for 4 days followed by Melphalan 140 mg/m^2^ IV in 250 mL of normal saline (dose reduction to 120 mg/m^2^ if SCr > 200 μmol/L) pre-transplant OR
(b) Carmustine 400 mg/m^2^ over 60 min (single dose) and Thiotepa 5 mg/Kg IV 12 hourly in 250 mL of normal saline for 2 days pre-transplant.

#### 2.2.2. Patients with Myelodysplastic and Myeloproliferative Disorders Were Broadly Classified under Other Haematological Conditions and Were Divided into Two Subgroups

(a) Patients who received reduced-intensity (non-myeloablative) allogenic HSCT with Fludarabine (30 mg/m^2^ IV over 30 min for 5 days) and Busulphan (3.2 mg/m^2^ over 30 min for 3 days) and/or Cytosine Arabinoside 2 g/m^2^ IV over 4 h for 4 days) followed infrequently by Melphalan (140 mg/ m^2^ in 250 mL of normal saline) or total body irradiation (TBI)-single fraction of 200 cGy/2 Gy.
(b) Patients who received full-intensity (myeloablative) allogenic HSCT with Lomustine (200 mg/m^2^ single dose), Etoposide (200 mg/m^2^ over 2 h in 1000 mL of normal saline) and Cytosine Arabinoside (200 mg/m^2^ 12 hourly over 30 min for 4 days followed by Melphalan 140 mg/m^2^ IV in 250 mL of normal saline and TBI fractions of 330 cGy for 3–4 days).

Post-transplantation immunosuppression was achieved by methotrexate (15 mg/m^2^) and calcineurin inhibitors, e.g., cyclosporine. Pre-transplant and post-transplant antibacterial, antifungal and antiviral prophylaxis were provided to all patients.

### 2.3. AKI Definition

AKI was defined and classified according to the Acute Kidney Injury Network criteria: Stage 1—increase in serum creatinine (SCr) ≥ 0.3 mg/dL (≥26.5 μmol/L) or increase of ≥150% to 200% (1.5- to 2-fold) from baseline; Stage 2—increase in SCr > 200% to 300% (>2- to 3-fold) from baseline; and Stage 3—increase in SCr > 300% (>3-fold) from baseline, or ≥4.0 mg/dL (≥354 μmol/L) with an acute increase of at least 0.5 mg/dL (44 μmol/L) or on renal replacement therapy (RRT) [46].

### 2.4. Study Measurements and Laboratory Analysis

Random urine samples (20 mL) were collected at 3 time points—at baseline or on the day of admission; Day 0, on the day of transplant; and Day 7, on the 7th day post-transplant. All participants were followed up, and any episode of AKI that occurred within a 30-day period from Day 0 was noted.

Ten millilitres from each urine sample collected was sent for analysis of the urinary creatinine concentration, protein concentration and protein-to-creatinine ratio. The analysis together with the analysis of all blood samples was collected at the same time for serum urea and creatine at the standardised NHS biochemistry laboratories of the participating centre.

The remaining 10 mL of the urine sample was centrifuged at 2400 rpm for 10 min at 20 °C and was stored at −80 °C. The samples were thawed overnight in batches in 2–8 °C refrigerators before being used for analysis. UL-FABP was measured semi-quantitively using a POC kit utilising the principles of lateral flow immune-chromatography assays, and its levels were quantified using the Renischem L-FABP ELISA TMB Kit using a 2-step sandwich method. All samples were assessed in duplicate within the same ELISA plate. The intra and inter assay variability for the particular assay was acceptable and previously published [47]. All L-FABP ELISA levels were corrected for the urinary creatinine concentration to compensate for differences in the urine flow rate. The levels used in the statistical analysis were the corrected levels. Both kits were supplied by CMIC HOLDINGS Ltd., Tokyo, Japan, and were used according to the manufacturer’s instructions. The POC test gave a score of ①, ②, ③ or ④ for the risk of developing AKI. ① corresponded to undetectable levels (0–12.5 mcg), ② represented uL-FABP levels of 12.5–100 mcg, ③ was associated with 100–400 mcg and ④ represented <400 mcg.

### 2.5. Statistics

The demographic and biochemical characteristics of participants were analysed using descriptive epidemiology. Categorical variables were presented as frequencies and percentages. Continuous variables with normal distributions were presented as means and standard deviations (SDs) while variables with skewed distributions were presented as medians with minimum and maximum values. The normality of the distribution was assessed using the Shapiro–Wilk method. A group comparison of baseline demographic characteristics between recipients of autologous and allogenic HSCT was performed using Pearson’s Chi-square for categorical variables and the Mann–Whitney U test for continuous variables. *p*-values > 0.05 were considered statistically significant. Receiver Operating Characteristic (ROC) curve analysis was used to evaluate the sensitivity and specificity of serum creatinine and uL-FABP measured through ELISA in detecting AKI at each time point, and the area under the curve (AUC) was reported. Univariate Logistic regression was used to assess potential confounders within the participants’ demographics, past medical history and biochemical profiles in predicting AKI up to 30 days post-transplant. Variables with statistical significance were used to build multivariate models of AKI prediction. Kaplan–Meier survival analysis was used to assess the hazard of mortality in the two HSCT groups. The performance evaluation of the POC tests against the uL-FABP ELISA for predicting a positive result was performed using Spearman’s correlation. Analysis was performed using the statistical software R 3.5.1 and Stata.SE 14.

## 3. Results

### 3.1. Patient Characteristics

A total of 85 patients were included in the study of which 65% were males. Patients’ demographic characteristics, study results and AKI status are summarized in Table 1. These were also stratified by the HSCT status (allogenic vs. autologous). 

### 3.2. Haematopoietic Stem Cell Transplantation and Acute Kidney Injury

Thirty-three participants (39%) experienced AKI during the follow-up period. The median time to AKI diagnosis was 25 [range 9–30] days. Participants who underwent allogenic HSCT were younger and had a higher incidence of Lymphoma and multiple myeloma but had a higher 30-day incidence of AKI and mortality. Median serum creatinine levels were fairly similar at baseline, day of transplant and on the 7th day post-transplant for both allogenic and autologous transplant patients. However, the median highest SCr within 30 days post-transplant was significantly higher in the allogenic group (121 µmol/L) than in the autologous group (96 µmol/L; *p* = 0.03) (Table 1). Corrected uL-FABP ELISA values at baseline, day of transplant and on the 7th day post-transplant were 1.61 mcg/gCr, 5.39 mcg/gCr and 10.27 mcg/gCr, respectively, for the allogenic group and 0.58 mcg/gCr, 4.36 mcg/gCr and 5.14 mcg/gCr, respectively, for the autologous group (Table 1). Table 2 summarises the uL-FABP (corrected) and SCr values at baseline, day of transplant and on the 7th day post-transplant stratified by the type of HSCT (allogenic or autologous) and the presence of AKI.

### 3.3. AKI Prediction Analysis

**Figure 1:**
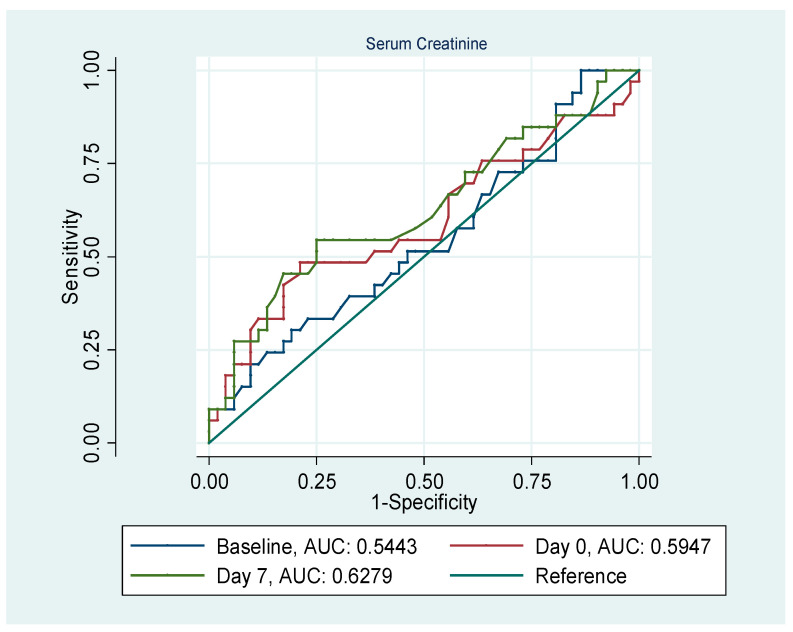
ROC curves for serum creatinine at baseline, day of transplant and on the 7th day post-transplantation in predicting AKI.

**Figure 2:**
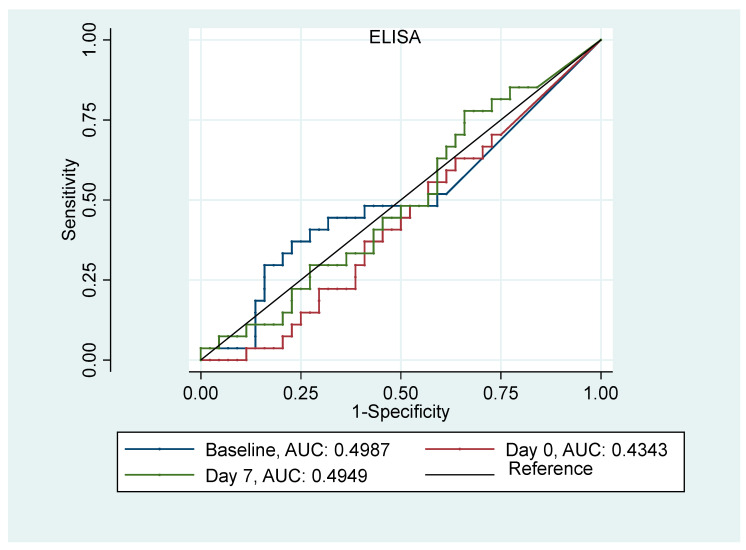
ROC Curves for uL-FABP at baseline, day of transplant and on the 7th day post-transplantation in predicting AKI.

The performances of SCr and uL-FABP (ELISA) in predicting AKI at different time points were evaluated using ROC curve analysis (Figure 1 and Figure 2). The AUC for both SCr serum and uL-FABP indicated that both these markers underperformed in predicting AKI, irrespective of the time point at which they were measured. The AUC was 0.54, 0.59 and 0.62, respectively, for baseline, day of transplant and 7th day post-transplant measurement for SCr and 0.49, 0.43 and 0.49 for uL-FABP. Univariate logistic regression analyses (Table 3) indicated that the risk of developing AKI was significantly increased in patients with allogenic transplantation (*p* = 0.004, 95%CI [0.1; 0.65]) and in those with creatinine clearance at baseline <30 mL/min or SCr > 200 mmol/L (*p* = 0.01, 95%CI [0.02; 0.54]). The risk of AKI was also significantly associated with the level of SCr on the 7th day post-transplant (*p* = 0.03, 95%CI [1; 1.03]).

Multi-variable logistic regression was used to determine the ability of the type of HSCT, renal impairment (creatinine clearance at baseline <30 mL/min or SCr > 200 mmol/L), ELISA uL-FABP and SCr to predict the occurrence of AKI (Table 4). Pre-existing renal impairment requiring a chemotherapy dose reduction was found not to be a significant predictor and was removed from the models. The type of HSCT was significant in all 3 models (Table 3). The risk of AKI was approximately 4 times higher in the allogenic group compared to the autologous group at all time points. The odds ratios for the HSCT type were 0.26 (95% CI [0.095, 0.67]; *p* = 0.006) at the baseline, 0.19 (95% CI [0.06, 0.53]; *p* = 0.002) on the day of transplant and 0.23 (95%CI [0.07, 0.72]; *p* = 0.015) on the 7th day post-transplant. UL-FABP was not shown to be an independent predictor of AKI in our population.

### 3.4. Survival Analysis and uL-FABP ELISA

Twelve patients died within 18 months after receiving stem cell transplantation, of which 10 (26%) patients were from the allogenic group and 2 (4%) were from the autologous group (*p* = 0.004). Median uL-FABP ELISA at baseline, on the day of transplant and on the 7th day post-transplant were slightly higher in those who died but the differences were not statistically significant. Cox proportional hazard regression analysis was performed to assess the effect of different variables in predicting the hazard of mortality. The type of HSCT (OR = 0.125; 95%CI [0.027; 0.57]; *p* = 0.007) was significant.).

### 3.5. Association of uL-FABP ELISA with POC

A semi-quantitative estimation of uL-FABP was performed using a POC device, and the relationship between POC and ELISA was explored with respect to whether POC uL-FABP scores (Table 5) corresponded to their mentioned uL-FABP ELISA levels. POC ➀ referred to L-FABP ELISA 0–12.5 mcg and POC referred to L-FABP ELISA > 12.5 mcg to 400 mcg. POC correlated with uL-FABP ELISA (*p* < 0.001, Spearman’s correlation = 0.54).

## 4. Discussion

This study set out to evaluate the potential of uL-FABP as a novel AKI biomarker and the performance of a POC test that could facilitate the utility of such a biomarker in clinical practice. This POC has been shown to be of value in predicting AKI in the Emergency Department setting [48], in trauma [38] and following emergency laparotomy [43]. To perform this evaluation, we chose a high-risk group of patients for developing AKI. With the incidence of AKI of up to 70% reported in HSCT recipients [45], we anticipated a high occurrence of this phenomenon. Nearly 40% of our participants did develop AKI within the first month after transplantation. The incidence of AKI was significantly higher in those receiving allografts compared to autologous grafts, consistent with the published literature [2,9]. Both groups of patients showed steady increases in SCr levels mirrored by the levels of uL-FABP. Interestingly, patients who received autologous HSCT had higher levels of both SCr and uL-FABP than those who received allogenic transplants, especially on day 7 post-transplantation. Not surprisingly, uL-FABP underperformed in predicting AKI in HSCT recipients at all stages, with only creatinine day 7 post-transplantation having a plausible predictive value. This was demonstrated in a multivariate model assessment. Arguably, the strongest predictor of AKI was undergoing allogenic HSCT despite the younger age of this group. These patients had a considerably different primary haematological condition profile. The majority of patients that received autologous transplants (88%) had multiple myeloma with only 6% of allogenic HSCT suffering from this condition. UL-FABP was included in this model but did not appear to have an independent predictive value. The extended median time of 25 days for developing AKI possibly indicates that the kidney injury advanced over a period of time, possibly due to the cumulative effect of therapeutics and intrinsic transplant biology combined together. This is similar to the results reported in the literature, with the average time to onset being 17 days and to maximum AKI severity being 30 days [2]

In a study of 84 HSCT recipients, Shingai et al. [45] found high levels of uL-FABP to predict AKI. The group consisted entirely of allograft recipients. Although their incidence of AKI was similar to ours (37% vs. 39%, respectively), they had a higher incidence of severe AKI (Stage 2/3 AKI 25 vs. 7) with a shorter median time to AKI (14 vs. 24 days). They also noted that important confounders to AKI development are advanced age and primary disease severity. Our cohort had unique characteristics of mixed types of HSCT and a lower incidence of severe AKI, perhaps explaining some variation between the findings. Interestingly, Shingai et al. did not report actual uL-FABP levels at baseline, but rather their analysis was based on dichotomising their cohort into detectable and undetectable uL-FABP using a cut-off level of 8.5 mcg, based on data from 412 healthy volunteers. Other studies that have assessed the uL-FABP POC device used cut-off levels of 12.5 mcg [38,43,48]. All but four of our participants had levels below these cut-offs at the start of the study.

There are several limitations in this study. The absence of a large number of patients in the detectable L-FABP range in the study limits the interpretation of our findings. The prolonged interval between the final measurement of uL-FABP and the median 25 days of incidence of AKI makes it possible that subsequent fluctuations in L-FABP levels may not have been captured. This study was conducted in the setting of a single large regional haematological referral centre for HSCT. This might also limit the generalisability of our findings, and further studies in multicentre settings with varied practice patterns would be helpful. Our attempt to compare the two biomarkers, SCr and uL-FABP, for AKI detection is also limited by the fact that, unlike SCr, the percentage of increase in uL-AFBP to define a clinically significant episode of AKI is unknown. We were not able to establish the correlation of the biomarker changes with histologic changes in the kidney biopsy, as the latter was not performed during the study, particularly with significant numbers in AKI stage 1, which do not warrant histological assessment. A longitudinal collection of biomarker data to extend into the follow-up period past the 7th day post-transplantation would yield data on the natural course and recovery patterns of uL-FABP in AKI.

Haemopoietic stem cell transplantation is a highly complex medical procedure often complicated by AKI, sepsis and increased mortality, particularly in patients receiving allogenic transplantation [3,49], and this was something observed in our cohort as well. AKI is a manifestation of severe systemic dysfunction, it complicates one out of five hospital emergency admissions [50] and it carries a mortality of 10% [51,52]. This can rise to 50% [53] in patients with AKI requiring Intensive Care Unit admission and to 80% if renal replacement therapy is required [54]. More than 65% of patients affected by AKI will recover renal function, although up to 10% will remain dialysis-dependent [55]. Furthermore, 20–30% of cases developing AKI can be prevented [50]. The speed of assessment is of paramount importance and, in this regard, POC uL-FABP assessment could be an important benefit in its utility. A useful finding in this study is the demonstration that POC test performance in detecting levels of uL-FABP < 12.5 mcg is comparable to that of ELISA with high sensitivity. However, the lack of overall utility limits the application of such rapid testing. POC assays will gain increasing traction for AKI management in high-risk pathways as they recently have been shown to be useful in predicting the development of AKI at the point of admission [48]. It is also unclear to what extent other factors such as deranged liver function could determine its value. uL-FABP has been shown to closely reflect GFR in CKD states [47]. Further studies are needed to determine the nature of its utility, alone or incorporated into prediction models, and whether different levels of uL-FABP can predict the severity of AKI and impact hospitalisation and mortality.

## 5. Conclusions

This prospective study evaluated the value of the novel AKI biomarker uL-FABP in predicting AKI in HSCT recipients. The utility of uL-FABP in the early detection and prediction of AKI in HSCT remains unclear. At all stages, the urinary L-FABP assay was unable to demonstrate an independent predictive value in our cohort. The uL-FABP POC assay correlated with ELISA measurements, but the low overall predictive value limits its utility. The most powerful predictor of AKI in this setting appears to be clinical characteristics such as the type of HSCT (allogenic). Further studies are required to assess whether the availability of the urinary POC AKI biomarker test (L-FABP) for rapid testing and screening can be utilised to potentially reduce the burden and severity and prevent AKI in HSCT.

## Figures and Tables

**Table 1:** Demographic characteristics, study results and AKI status.

*n* = 85	Allogenic HSCT *n* = 35 (41.2%)	Autologous HSCT *n* = 50 (58.8%)	*p*-Value
**Age** median (IQR) [range] 62 (56, 68) [24–75]	59 (48, 67) [24–70]	63 (59, 69) [32–75]	<0.001
**Gender** *n* (%)			
Female, 30 (35%)	8 (23%)	22 (44%)	0.076
Male, 55 (65%)	27 (77%)	28 (56%)	
**Ethnicity** *n* (%)			
White, 78 (95%)	33 (94%)	45 (90%)	0.350
Asian, 4 (5%)	1 (3%)	3 (6%)	
Black, 2 (2%)	-	2 (4%)	
Other, 1 (1%)	1 (3%)	-	
**Primary Haematological Condition** *n* (%)			
Lymphoma, 6 (7%)	2 (6%)	4 (8%)	<0.001
Multiple Myeloma, 46 (54%)	2 (6%)	44 (88%)	
Other, 33 (39%)	31 (88%)	2 (4%)	
**Medical History** *n* (%)			
Diabetes, 12 (14%)	3 (9%)	9 (18%)	0.360
Hypertension, 32 (38%)	10 (29%)	22 (44%)	0.220
CKD, 12 (14%)	2 (6%)	10 (20%)	0.120
**ACE inhibitor**			
Ramipril, 10 (12%)	3 (9%)	7 (14%)	
Lisinopril, 5 (6%)	2 (6%)	3 (6%)	
Perindopril, 1 (1%)	1 (3%)	-	
**Kidney Function Status pre-HSCT** *n* (%)			
Creatinine Clearance > 30 mL/min 75 (88%) Creatinine clearance < 30 mL/min 10 (12%)	28 (80%) 7 (20%)	47 (94%) 3 (6%)	0.130
**Creatinine μmol/L** median (IQR) [range]			
Baseline	87 (75, 101) [58–263]	80 (66, 103) [44–258]	0.390
Day of transplant	71 (59, 100) [8–258]	74 (63, 94) [40–273]	0.460
Day 7	75 (63, 4) [36–230]	77 (2, 104) [38–329]	0.530
Day 30	96 (78, 129) [50–210]	89 (70, 110) [44–288]	0.160
**Highest Serum Creatinine μmol/L** median (IQR) [range]	121 (98, 159) [57–310]	97 (85, 131) [52–449]	0.033
**Uncorrected uL-FABP ELISA mcg** median (IQR) [range]			
Baseline 0.5 (0, 2.8) [0, 126.2]	0.8 (0, 3.1) [0–62.1]	0.2 (0, 2.0) [0–126.2]	0.310
Day 0 2.1 (0.1, 7.3) [0, 262.4]	2.2 (0.7, 7.2) [0–173.1]	2.1 (0, 8.5) [0–262.4]	0.300
Day 7 6.4 (1.8, 15.5) [0, 79.5]	6.9 (2.4, 18.5) [0–79.5]	5.5 (1.3, 10.2) [0–68.8]	0.180
**Corrected uL-FABP ELISA mcg/gCr** median (IQR) [range]			
Baseline 0.6 (0, 5.4) [0, 96.3]	1.6 (0.1, 3.0) [0–96.3]	0.6 (0, 6.4) [0–70.3]	0.480
Day 0 5.1 (0.7, 17.1) [0, 662.8]	5.4 (2.0, 10.1) [0–49.0]	4.4 (0, 18.0) [0–662.9]	0.980
Day 7 5.9 (2.0, 16.5) [0, 125.5]	10.3 (3.2, 23.0) [0–125.5]	5.1 (1.1, 11.8) [0–47.1]	0.041
**AKI in the first 30 days, n (%)**	20(57%)	13(26%)	0.008
No AKI AKI score = 1 AKI score = 2 AKI score = 3	15 14 5 1	37 12 0 1	
Mortality 18 months post-HSCT, n (%)	10 (26%)	2 (4%)	0.004

**Table 2:** uL-FABP and serum creatinine levels at different time points stratified by HSCT type and the presence of AKI.

	Allogenic HSCT			Autologous HSCT		
	No AKI	AKI	*p*-Value	No AKI	AKI	*p*-Value
**uL-FABP ELISA mcg/gCr,** **Median (IQR) [range],**						
**Baseline**	1.6 (0.3, 2.2) [0–30.5]	0.8 (0, 3.0) [0–96.3]	0.470	0.2 (0, 2.6) [0–70.3]	2.8 (0, 7.7) [0–12.2]	0.760
**Day of Transplant**	5.5 (1.7, 16.1) [0–49.0]	4.1 (1.6, 8.4) [0–34.7]	0.690	4.0 (0, 17.1) [0–101.8]	4.4 (0, 14.9) [0–77.2]	0.720
**Day 7th post-transplant**	14.6 (4.8, 38.2) [0–74.1]	5.1 (3.0, 20.2) [0–125.5]	0.370	3.5 (0, 9.8) [0–47.1]	6.4 (2.2, 9.2) [0–19.7]	0.720
**Serum Creatinine μmol/L** **Median (IQR) [range]**						
Baseline	94 (80, 101) [59–132]	81 (73, 104) [58–263]	0.590	79 (67, 100) [44–225]	93 (65, 140) [58–258]	0.340
Day of Transplant	68 (58, 88) [49–102]	78 (63, 105) [41–258]	0.220	73 (63, 92) [40–203]	94 (70, 143) [57–273]	0.090
Day 7th post-transplant	72 (63, 77) [36–94]	86 (61, 100) [44–230]	0.130	76 (55, 92) [38–228]	99 (68, 130) [60–329]	0.073

**Table 3:** Uni-variable analysis of potential AKI risk factors.

Variable	Odds Ratio (95% CI)	*p*-Value
Log (Baseline uL-FABP ELISA)	1.01 (0.85,1.19)	0.90
Log (uL-FABP ELISA Day 0)	1.004 (0.85,1.29)	0.97
Log (uL-FABP ELISA Day 7 *)	1.06 (0.87,1.31)	0.62
Baseline SCr	1.01 (1, 1.019)	0.21
Day 0 SCR	1.01 (1, 1.024)	0.07
Day 7 SCR	1.02 (1, 1.033)	**0.03**
**Graft type**		
Allogenic	1	**0.004**
Autologous	0.26 (0.1, 0.651)	
**Condition**		
Multiple Myeloma	1	0.09
Other	2.17 (0.9, 5.364)	
**Sex**		
Female	1	0.09
Male	2.29 (0.89, 6.304)	
**CKD**		
No	1	0.14
Yes	2.53 (0.74, 9.319)	
**Diabetes**		
No	1	0.83
Yes	1.15 (0.31, 3.949)	
**Hypertension**		
No	1	0.79
Yes	1.13 (0.46, 2.771)	
**Creatinine clearance at baseline <30 mL/min or SCr > 200 mmol/L**		
Yes	1	**0.012**
No	0.13 (0.018, 0.54)	

* Day 7 was considered only for those where their highest SCr was observed after day 7. *p*-values that appear in bold are statistically significant at the level of *p* < 0.05.

**Table 4:** Multi-variable logistic regression of uL-FABP and other risk factors in predicting AKI.

Variables in the Model	Odds Ratio (95% CI), *p*-Value for uL-FABP	Odds Ratio (95% CI), *p*-Value for Graft Type	Odds Ratio (95% CI), *p*-Value for SCr
Baseline uL-FABP, Graft type, Baseline Sc	0.94 (0.76, 1.14) 0.53	0.26 (0.095, 0.67) 0.006	1.009 (0.99, 1.02) 0.15
Day 0 uL-FABP, Graft type, SCr Day 0	0.86 (0.69, 1.06) 0.17	0.19 (0.06, 0.53) 0.002	1.02 (1.01, 1.04) 0.011
Day 7 * uL-FABP, Graft type, SCr Day 7	0.98 (0.78, 1.25) 0.87	0.23 (0.07, 0.72) 0.015	1.02 (1.01, 1.05) 0.019

SCr = serum creatinine * Day 7 was considered only for those where their highest SCr was observed after day 7.

**Table 5:** Performance evaluation of POC against ELISA.

	ELISA <12.5	ELISA ≥12.5	Sensitivity, 95% CI	0.99 (0.97, 1)
POC = 1	227	17	Specificity, 95% CI	0.65 (0.49, 0.78)
POC ≠ 1	2	31	Positive Predictive Value, 95% CI	0.93 (0.89, 0.96)
			Negative Predictive Value, 95% CI	0.94 (0.8, 0.99)

## Data Availability

Data cannot be made publicly available until the completion of all work relating to this study.
